# Water Disinfection Systems for Pools and Spas: Advantages, Disadvantages, and Consumer Views in the US

**DOI:** 10.1021/acsestwater.4c00612

**Published:** 2025-01-09

**Authors:** Mourin Jarin, Jackie Ly, Jonathan Goldman, Xing Xie

**Affiliations:** † School of Civil and Environmental Engineering, Georgia Institute of Technology, Atlanta, Georgia 30332, United States; ‡ Office of Commercialization, Georgia Institute of Technology, Atlanta, Georgia 30332, United States

## Abstract

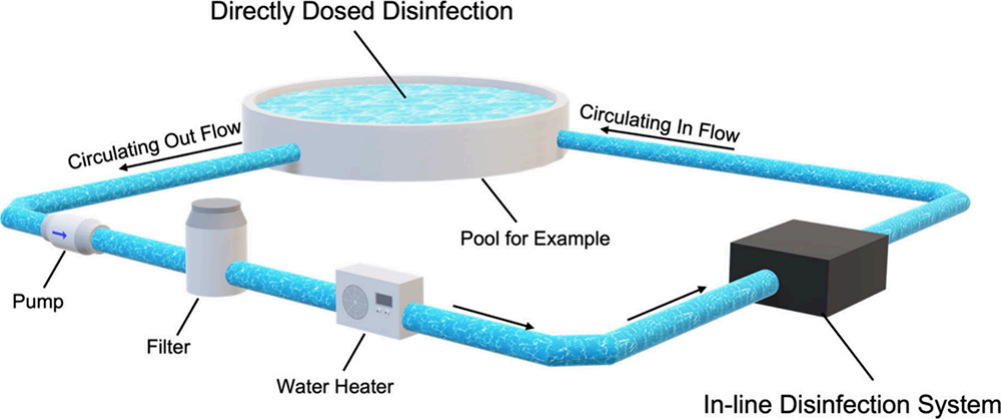

Disinfection of swimming pools and hot tubs (pools/spas) are necessary to prevent outbreaks and exposure to waterborne pathogens from water recreation. However, harmful disinfection byproducts (DBPs) from heavy chlorine usage continue to be a growing concern. Chlorine-based disinfectants also react with human inputs like sweat, urine, cosmetics, sunscreen, etc., that are introduced in a pool/spa, further increasing the severity of the DBP problem. We reviewed the current status of water disinfection technologies in the pool/spa industry and summarize the methods, trends, advantages, and disadvantages from a health and consumer viewpoint. Market research and face-to-face interviews were also accomplished with 100 industry experts and end-users in the US. We then integrate the literature findings in parallel with these market insights. Overall, we conclude the future of water recreation is trending away from high dosage chlorine-based solutions to disinfect swimming water and turning to alternatives with better sustainability and safety in mind. Lastly, we discuss the future directions of these technologies with current and past trends, offering insights to where research and development should be focused for both the user’s health and overall experience.

## 1 Introduction

For the past 100 years, chlorine has been used in municipal water disinfection to protect people from waterborne infections. Disinfection of swimming pools and hot tubs (pools/spas) are also necessary to prevent outbreaks and exposure to waterborne pathogens from water recreation.^1^ This has traditionally been done by dosing chlorine into the swimming water (pool/spa water that people can actively swim/bathe in).^2^ However, over the last 50 years, we have discovered that these standard chlorine-based disinfection methods contribute to the formation of harmful disinfection byproducts (DBPs).^3^ DBPs are formed when disinfectant (typically chlorine in this case) reacts with natural organic matter or inorganic substances present in the water.^4^ Researchers have associated exposure and consumption of these DBPs in swimming water to cancers, allergies, respiratory issues, and reproductive implications. Several additional studies have come out in the last two decades highlighting the elevated levels of DBPs found in water samples taken from pools/spas.^5−8^ Because chlorine-based disinfectant reacts with human inputs (sweat, urine, cosmetics, sunscreen, etc.), this increases the severity of the DBP problem.^6,9,10^

In the US, there are over 300 million visits to a swimming pool each year, with 36% being children and teens (age 7–17).^11,12^ Additionally, 15% of adults swim at least six times every year as swimming is the fourth most popular recreational activity, and the most popular among children and teens.^13^ According to the Pool and Hot Tub Alliance (PHTA), there are a total of 10.7 million swimming pools in the US recorded in 2023.^14^ Almost all of those (10.4 million) are private pools like those in people’s homes, while only about 300,000 are public pools. Additionally, there are also more than 7.3 million hot tubs in the US today.^14^ Despite the consistent popularity and growth of the water recreation market, the Center for Disease and Control (CDC) in the US has reported thousands of immediate closures and violations for public aquatic venues.^15^ Specifically, a study of 84,187 routine inspections for 48,632 public aquatic venues in five states resulted 12.3% to be immediately closed due to at least one identified public health threat/violation.^15^ The study further revealed disinfectant concentration violations were reported in 11.9% of routine inspections, representing a risk for outbreaks and infectious etiology. Pool chemical safety violations were identified as 4.6% of routine inspections, meaning a risk for pool chemical associated health events were present. Regarding public spas, CDC conducted a study of spa inspections from six states and found that out of 5,209 inspections a total of 5,378 violations were documented.^16^ More than half the inspections resulted in one or more violations, 11% resulted in immediate closures of the spas, 50.7% had water chemistry violations, and the highest record of violations occurred consistently in campgrounds and hotels/motels.

With the number of people engaging in water recreation every year and more and more children at a young age who spend time in pools/spas, it is becoming very important to assess the health risks and growing concerns for disinfection and chemical usage in this industry. Also, when we look toward the number of adolescents who start swimming from a young age, the concerns for exposure to carcinogenic DBPs from heavy chemicals have become more threatening.^17−19^ The first reported publication that discussed mutagenicity of swimming pool water was in 1980, and now there are thousands of articles related to these topics.^20^ Alarmingly, several studies have linked adolescent exposure to chlorine swimming pools with having a significant contribution to the development of asthma and respiratory diseases in children, while exposure to alternative disinfection in pools resulted in no such health impacts.^19,21−24^ It is important to note, these DBPs are not only present in the pool/spa water, but also in the in air surrounding indoor swimming pools/spas, increasing the exposure and intake through ingestion, inhalation, and dermal absorption.^7,17,25^ Due to the high levels of DBPs found in pools/spas caused by heavy chemical disinfectant usage, it is becoming more important to investigate alternatives for disinfection of swimming water. Recent investigations have shown promise for existing alternatives including electrochemical (salt systems), ultraviolet light (UV), ozonation (ozone), antimicrobial metals (Cu/Ag/Zn), and mixed methods that use several solutions together.^26−29^

Despite the rise in alternatives for disinfection, the standard chlorine-based treatment remains the most popular. Due to the challenges and growing concerns of heavy chemical usage and DBP exposure, there is increasing interest in the research and development of alternative disinfection methods for the expanding market of water recreation. In this perspective, we review the current status and operation of water disinfection technologies in the industry of pools/spas and summarize the methods, trends, advantages, and disadvantages from a health and consumer viewpoint. Market research on various positions of the water recreation business ecosystem was accomplished through face-to-face interviews with 100 individuals in the US. We then integrate the literature findings in parallel with these market research interviews in the pool/spa industry. We report these findings along with emerging technologies that could pave way for newer and safer disinfection in this field. Finally, we discuss the future directions of these technologies with current and past trends, offering insights to where research and development should be focused for both the user’s health and overall experience.

## 2 Current Solutions

An extensive review of the six most commonly administered disinfection technologies used in pools/spas today was accomplished and summarized in the following sections. These include chlorine, bromine, salt systems, UV, ozone, and antimicrobial metals. A brief comparison of all six methods and their contribution to the swimming water is provided in Table 1.

**Table 1:** Chemical Contributions of 6 Different Pool/Spa Disinfection Technologies to the Swimming Water

Disinfection Systems	Typical Dosage Range^a^	Free Cl Demand Reduction	DBP Contribution^b^	Effective against common Cl resistant microorganisms
Chlorination	1–3 mg/L^30^ (free chlorine)	NA	Contributes heavily^31,32^	Not effective^33^
Bromination	3–4 mg/L^30^ (free bromine)	100%	Contributes heavily^10,34,35^	Not effective^36^
Salt System	3000–5000 mg/L^27^ (sodium chloride)	∼50%^27^	Contributes heavily^27,28,37^	Not effective^33^
Ozonation	0.8–1.5 mg/L^38^	40–80%^39,40^	Contributes to reduction^28,41,42^	Effective^40,43^
Ultraviolet Radiation	∼1.34 kW h m^–3^ d^–1^ ^44,45^	50–80%^39,46^	Contributes to reduction^29,47−49^	Effective^43,50^
Antimicrobial Metals	<0.6 mg/L Cu^51,52^	0–90%^39^	Contributes heavily to reduction^26^	Slightly effective^43,53^
	<0.03 mg/L Ag^39^			

a The typical dosage range for each disinfection system refers to the concentration required in a pool/spa to achieve optimal disinfection.

b DBP contribution of each disinfection system is respective to an already chlorinated/brominated pool/spa (each system is acting as an additional disinfection mechanism to a typical and already disinfected pool/spa). For further reference to the specific DBPs, their formation potentials, and/or toxicity regarding each disinfection technique please refer to the cited review by Li et al. focused on DBP formation in swimming pools across the globe.^2^

### 2.1 Chlorine

**Figure 1:**
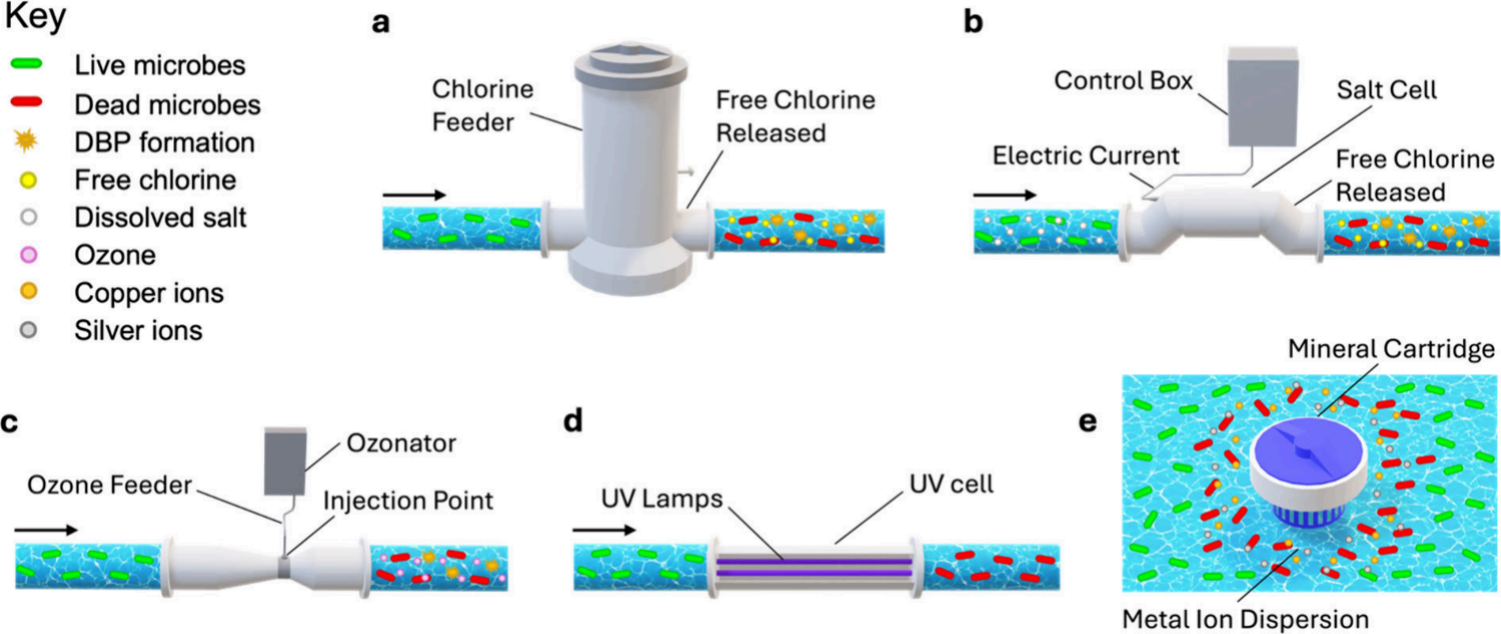
**Schematic showing typical flow operation and disinfection for 5 different pool/spa technologies.** The 5 main categories of disinfection are visualized as (a) chlorine through a tradition chlorine feeder, (b) salt system using a salt cell, (c) ozone dosed through an ozonator, (d) UV operated through a UV cell, and (e) antimicrobial metals released through a mineral cartridge. A key is shown to guide the reader on the many different disinfection agents involved in each process. Bromine is not represented here as it is uncommonly used for pools. (For any reference to color in the figure, the reader is referred to the online/web version of this article).

Chlorine has been used as a standard disinfectant for pools/spas extensively, remaining the ubiquitous and most widely recognized disinfection option.^7^ Most swimming water relies on the effectiveness of free chlorine, specifically hypochlorous acid (HOCl) and hypochlorite ions (OCl^–^), as the strong oxidant to prevent and kill pathogens.^54^ Because most spas and many pools generally have higher water temperatures, are exposed to sunlight, and have high organic loads, the rate of chlorine decay increases along with chloramine formation, resulting in the free residual chlorine demand remaining very high.^7^ Pools/spas compensate for this by maintaining high doses of disinfectant to ensure there is free chlorine residual always available.^55^ As a result, the standard in the US that CDC recommends is maintaining a pool/spa within a pH of 7.2–7.8, and a free chlorine concentration of at least 1 mg/L in pools and 3 mg/L in spas.^30^ Comparatively, the free chlorine residual in drinking water in the US is typically between 0.2 and 0.5 mg/L, with the minimum goal of 0.2 mg/L and a maximum residual disinfectant level set by the US Environmental Protection Agency (EPA) at 4 mg/L.^56^ Chlorine is typically administered in pools/spas through a chlorine feeder (Figure 1a). The several types of chlorine used for disinfection vary in form and include sodium hypochlorite, calcium hypochlorite, chlorine gas in indoor pools, and stabilized solid chlorine products for both indoor and outdoor pools.^7,57^ These are most commonly referred to as liquid bleach (sodium hypochlorite), dichlor (granular stabilized chlorine), trichlor (stabilized chlorine in tablets, pucks, and sticks), and calcium hypochlorite (granular and can be stabilized/unstabilized depending on usage).^54^ For further details regarding the reaction mechanisms for chlorine disinfection in a pool/spa please refer to the paper by Tsamba et al.^58^

A critical advantage chlorine has in the disinfection of pools/spas is its ability to perform shocking. Shocking a pool/spa refers to adding a large dosage of chlorine disinfectant at one time (rapidly increasing the free chlorine concentration) in order to reset the pool from a high accumulation of organic/inorganic contaminants or to quickly inactivate any potential threat of infection that lies in the pool.^54,59,60^ Typically, the dosage of chlorine required is ten times the current concentration of combined chlorine/chloramines present in the swimming water.^54^ Shocking is necessary specifically for public facilities where there are often children who may urinate/defecate/vomit in the pool, thus requiring an immediate need for evacuation.^54^ This very important process allows a pool/spa to continue to be active a few hours or one day after shocking for a quick disinfection turnaround. There exist still some drawbacks to shocking including the large amount of chlorine required, potential damage to liners and swimsuits, disturbing the water chemistry/balance, difficulty to determine a proper dosage, and inability to use the pool/spa until chlorine levels drop to a respectable and safe 1–4 mg/L.^54^ Despite this, the shock method still provides the fastest form of reviving a pool/spa for continued use. This ability to shock is something that we have identified to limit the transition or addition of alternative disinfection methods to large/public pools as chlorine is still the top choice if any incidents occur, and alternatives cannot be relied on as much in these cases. Generally, for smaller water bodies like in hot tubs, there is less importance placed on shocking as it is easier to treat and reset a smaller volume of water for less people with less overall risk.

Besides the advantages that allow chlorine to be the standard disinfectant used across the world in water recreation, there are still many disadvantages as well. First, chlorine-resistant parasites like *Cryptosporidium*, most commonly associated with pathogen outbreaks from swimming pools, can survive for 3.5–10.6 days in water maintained the recommended chlorine levels (1–3 mg/L).^33^ Beyond this, due to the continuous heavy disinfection and high organic load from people leaving and entering, pools/spas have been recognized as high DBP environments both in the water and in the air.^32^ Many factors contribute to the formation of more and more DBPs in pools/spas including the chlorine dosage, free residual chlorine, temperature, organic loading, contact of the swimming water with the air, and the overall water recirculation.^7^ Today more than 700 DBPs have been identified in swimming waters.^31^ Researchers have extensively studied many of the DBPs identified in pools/spas and over 100 have been revealed to be genotoxic and more than 20 are carcinogenic.^61−63^ More than 100 of these were identified in only pools/spas, while not found in typical drinking water treated with chlorine, specifically DBPs containing nitrogen, which are formed from the sweat and urine highly present in pools/spas.^9,64,65^ Lastly, extended time and exposure to chlorinated pools/spas have been shown to cause common and repeat symptoms of itchiness, eye irritation, skin irritation, asthma, etc.^5,7,66,67^ For any further reference to the specific DBPs, their formation potentials, and/or toxicity regarding each disinfection technique please refer to the cited review by Li et al. focused on DBP formation in swimming pools across the globe.^2^

### 2.2 Bromine

Bromine is also a common chemical used similarly to chlorine for its powerful oxidizing capabilities in swimming water disinfection. The main oxidizing agent that gives bromine its disinfection ability is the formation of bromine into hypobromous acid (HOBr).^6,10^ This chemical is less dependent on pH compared to chlorine and remains a strong disinfectant even after turning into combined bromine from interaction with contaminants. The CDC recommends a free bromine concentration of at least 3 mg/L in pools and 4 mg/L in spas.^30^ It is not used as widely as chlorine but has become more and more popular in spa disinfection specifically to treat the hot and turbulent water which dramatically increases the accumulation of organic/inorganic contaminants in the tub.^54^ The accumulation of contaminants in spas places heavy stress on chlorine, forming increased concentrations of combined chlorine/chloramines which produce odor and irritation. Bromine as a disinfectant does not suffer from this problem, and therefore has gained popularity as a common chemical used in spas. Bromine is generally available in similar forms to chlorine including tablets, sticks, caplets, and in two product systems.^54^ These are typically applied through various types of inline feeders or floating feeder devices (similar to chlorine), although when using sodium bromide salts, an oxidizer of chlorine can act as the trigger to convert the salt into free bromine. For further details regarding the reaction mechanisms for bromine disinfection in a pool/spa please refer to the paper by El-Athman et al.^68^

The most prominent advantage to bromine usage over chlorine is its lack of eye and skin irritation, along with no foul odor produced.^54^ Despite this, there are some limitations with bromine along with very pressing concerns regarding its role in DBP formulation. Bromine (along with chlorine) is ineffective to common parasites like *Cryptosporidium* in water maintained at the recommended levels, leaving pools/spas vulnerable to common pathogen outbreaks.^36^ Studies have also shown pools and spas that use bromine as a disinfectant are generally found to produce more mutagenetic, genotoxic, and cytotoxic DBPs than chlorine.^10,34,35^ A study looking at chlorinated versus brominated pools found that brominated pools with Br-DBPs were almost twice as mutagenic.^10^ Another study found brominated pools could be up to 30 times higher in toxicity due to the various classes of Br-DBPs.^69^ Despite the advantages, the negative effects for bromine quickly outweigh chlorine as the previously mentioned studies show bromine-treated water in both pools and spas to be more mutagenic and toxic, providing increased health risk for users (any swimmers, bathers, owners, consumers, etc.) compared to traditional chlorine products.

### 2.3 Salt Systems

In 2021, a national chlorine shortage occurred in the US due to a number of reasons including the closure of two main manufacturing facilities and limitations to operation during the COVID-19 pandemic.^70^ This greatly impacted the ability to use chlorine to disinfect pools/spas across the country due to the limited supply and spiked prices. During this time, existing alternative disinfection techniques, specifically electrochemically generated chlorine (salt systems/saltwater pools) became much more popular as they could provide chlorine to consumers who otherwise had no way of accessing it for their pools. These salt systems generally work by passing an electric current through a slightly concentrated salt solution (typically 3,000–5,000 mg/L).^27^ The current is applied by the installed system and the flowing saltwater is the swimming water with the addition of sodium chloride.^27^ Because the swimming water is salted, these pools are often called saltwater pools in the US, not to be confused with pools that are filled with seawater or salted water not converted into chlorine. The salt systems electrochemically oxidize the saltwater to produce hypochlorous acid (HOCl) and hypochlorite ions (OCl^–^) as the main oxidants for disinfection (Figure 1b).^71^ For further details regarding the reaction mechanisms for salt water systems please refer to the paper by Granger et al.^27^

Salt systems had already been growing in popularity for the past decade as the investment costs for the equipment and installation were reduced significantly.^27,72^ Despite this, it is still known to be one of the most expensive options using chlorine when all the upfront and long-term costs are totaled.^72^ The main advantage for users (any swimmers, bathers, owners, consumers, etc.) is the minimized maintenance required. As the salt systems involve adding salt into the pool rather than chlorine or other chemicals, it is slightly safer and simpler for the user as well. Because the salt system can maintain a chlorine concentration in situ, it is often less than a typical pool/spa and can help to reduce the residual free chlorine concentration needed for safe swimming. A study comparing pools using either liquid sodium hypochlorite or electrochemically generated chlorine showed the latter to have 50% or less of the free residual chlorine concentration while maintaining the same function.^27^ Unfortunately, the constant chlorine output into the pools still remains a concern for potential chlorine-resistant pathogens and the production of harmful DBPs. Although there are still limited studies on the differences of DBPs in traditional chlorine or saltwater pools, there are already some increasing concerns. A study on 60 different DBPs found that saltwater pools measured a 15% increase in the total concentration of DBPs generated compared to traditional chlorine pools, but the overall cytotoxicity and genotoxicity of these DBPs decreased by 45% and 15%, respectively.^27^ Saltwater pools were also found to have 70% higher levels of bromine-induced DBPs in comparison to traditional chlorine pools due to the bromide impurities present in the salt administered to the pools.^27,28,37^ The bromide impurities leading to increased toxicity of the swimming water is why many emphasize the importance of using high purity sodium chloride for their saltwater systems.^28,73,74^

### 2.4 Ozonation (ozone)

Ozonation (ozone) was first administered in swimming water treatment in 1964 and has since grown to be widely used in pools/spas.^38^ In the past 50 years, Europeans have been using ozone in their swimming pools; totaling around 300 million users today.^40^ Since then, the number of pools/spas using ozone for disinfection has been steadily growing in the US. Currently, most spas manufactured in the past 10 years have included an ozonator.^40,54^ Ozone is a strong oxidizing agent, and when applied to swimming water the ozone can oxidize dissolved organic carbon (DOC) and other pollutants.^75^ This reduction of dissolved pollutants also reduces the reactivity allowed for chlorine present in the water and therefore the overall DBP formation as well. Because of this, ozone has gained acceptance over the years as a potential precursor to chlorine. Chlorine is also typically applied to swimming water regardless due to the instability of ozone, short half-life, and the high dosage required for it to be a primary disinfectant.^28,76,77^ For further details regarding the reaction mechanisms for ozone disinfection either on its own or in combination with other chemical disinfection approaches please refer to the paper by Rice.^78^

Globally, most ozone treatments in pools/spas are still accomplished according to the German standard for swimming called the DIN 19643 (Deutsche Industrie Norm, also translated to German Industry Standard) as the federal standard for the “treatment and disinfection of swimming and bathing pool water,” where the contact time of ozone with swimming water was reported to be 3–10 min (long contact time) for the typical ozone concentrations used (0.8–1.5 mg/L).^38,79,80^ This 3–10 min contact time is traditionally followed by an activated carbon filter to destroy and prevent any active ozone remaining from escaping through the swimming water and into the air where it could be inhaled.^75,81^ Unfortunately, this results in a highly inefficient usage of the dosed ozone. In the US, a different process is also used called the slip-stream approach (short contact time with only part of the circulating water stream) where low-dose ozone is applied into a side stream and consumed rapidly by reacting with the organic matter present.^75^ This process controls the dosing using a redox probe to ensure the ozone is not added in excess. A typical ozone dosage in the US ranges between 0.4 and 0.8 mg/L.^40^

The first and most common method for ozone generation is called the corona discharge (CD) and is used in more than 90% of ozonators today.^40^ CD involves using two electrodes to create an electric field that ionizes the oxygen molecules in the air and reforms it into ozone, after this occurs in a control box, the ozone is released into the swimming water in a controlled dose (Figure 1c). CD generators tend to be more energy efficient compared to other methods as they produce higher concentrations of ozone at faster rates, leading to lower overall costs, despite this, the CD method also produces more impurities such as other gases though its process.^40^ Another method gaining popularity is UV-generated ozone, which uses UV radiation to photochemically break down oxygen molecules that then reform into ozone.^40^ This method produces a low concentration of ozone; therefore, it is less suitable for applications that require high doses. Regardless, the strong oxidizing power of ozone has made it a popular technology that continues to be implemented in various methods for swimming water disinfection.

Ozone is also implemented in combination with other processes like bromide and UV disinfection. A method exists to use both ozone and bromide in a single system where swimming water containing bromide is oxidized using an ozonator, producing HOBr which then acts as the primary disinfectant.^82^ In these ozone+bromide systems, key concerns like the produced ozone consumption or bromate formation can be avoided with high concentrations of bromide present in the water.^83^ Some systems combine UV and ozone independently (UV+ozone) to provide two layers of disinfection and can even be installed in hot tubs to operate without the use of any other additional chemicals.^84^ Ozone systems have a fairly long service interval (18–24 months) without needing any maintenance or repair.^40^ Despite this, UV lamps still require yearly replacement, making the combination systems a little less convenient. Ozone also has the ability to disinfect certain chlorine-resistant pathogens, such as *Legionella* and *Cryptosporidium*.^40,43^ We are now more commonly seeing ozone systems installed into spas as they help with the disinfection of the water and reduce the chlorine demand by 40–60%, typically.^40^ They break down particles and debris that accumulate in the tub, making the water less turbid. The major advantages of ozone are the strong disinfection capabilities, fast disinfection, and reduction of high chemical demand, reducing the harmful DBPs and health effects from chlorine.

Despite the many upsides with ozone, there are still some remaining concerns. It is expected that ozone can reduce organic pollutants in the swimming water, decreasing the subsequent chlorine reactivity with pollutants, leading to a decrease in DBP formation.^41^ Despite this, a disadvantage can include ozone decomposing into hydroxyl radicals if not quickly consumed. Hydroxyl radicals can react with organic matter and have been shown to increase the reactivity of chlorine to form DBPs by introducing more oxygen containing functional groups and leaving more compounds available for oxidation by chlorine.^41,45^ Some studies have shown ozone can oxidize precursors for chlorine formulated DBPs, minimizing one issue, but then form other uncommon byproducts potentially of concern.^85,86^ Other studies on ozone/chlorine treatments for pools/spas found either a decrease in the levels of DBPs compared to chlorinated pools or observed no difference at all.^28,42^ Another study specific to the DBPs found in ozonated swimming pools discovered decreased levels when ozonating a polluted pool, increased levels when low doses were applied to a clean pool, and decreased levels when repeated/high doses were applied.^75^ The authors concluded the ozone dosage in swimming water should be proportional to the water quality, but this is not common procedure currently in the pool/spa industry. In larger scale water treatment systems, control and measuring devices can be combined with chemical dosing of ozone to allow for disinfection accordingly to the degree of contamination of the water, but again, this is not standard for the US pool/spa industry where it is more common to rely on a constant dosage system.^87^ Specific to other ozone combined methods like UV+ozone, studies show promising results to both decrease the chlorine reactivity for byproduct formation and improve the overall chlorinated swimming water quality.^85^ A synergy has even been formulated that ozone may remove any unwanted UV-formed reactivity, and the UV photolyzes any ozone induced DBPs.^86^

### 2.5 Ultraviolet Light (UV)

UV has been around for the last century as a high inactivation source and has also been used specifically for water disinfection. UV light is a segment of the electromagnetic spectrum from 100 to 400 nm range. The short wavelengths between 220 and 300 nm are germicidal with the most damage to a cell caused at 265 nm.^88^ Because of this, low-pressure mercury lamps emit a common light of 254 nm to serve as a highly efficient disinfection source.^89^ The main mechanism for the inactivation of microbes is the UV light’s damage to the nucleic acid of the cells. As the UV light is absorbed, the genetic material inside the cells is altered, preventing DNA from replicating and leading to lethal and mutagenic effects, and, finally, cell death.^90^ Despite UV’s stand-alone disinfection capabilities that do not form any harmful DBPs, it does not provide any residual disinfection or strong oxidizing power (Figure 1d).^29,89^ Because of this, the implementation of UV into pools/spas is not typically on its own, but in combination with other approaches using strong oxidizers or oxidants. For further details regarding the reaction mechanisms for UV in combination with other disinfection methods please refer to the paper by Guo et al.^91^

The most common combination approaches include UV with chlorine (UV+Cl_2_), UV+ozone, and UV with hydrogen peroxide (UV+H_2_O_2_). UV+Cl_2_ is typically the traditional chlorination of UV-treated water. This is done with the main goal of reducing the total DBP concentration, specifically combined chlorine/chloramines which contribute to the irritation of eyes and upper respiratory, leading to lung damage, and increased asthma in children and lifeguards.^19,50,85,92−95^ UV+ozone is the combination of UV for inline disinfection and ozone for providing a strong oxidizing agent. Studies show UV can decrease the reactivity of contaminants in swimming water for both chlorine and ozone. UV used as a pretreatment to ozone can thus reduce the reactivity of any subsequent ozone and chlorine, decreasing the overall DBP formation, and improving the chlorinated water quality.^85^ A few other studies have also shown success eliminating the need for chlorination altogether; achieving disinfection with only the combination of UV+ozone and prefiltration.^84^ Lastly, UV+H_2_O_2_ offers a similar method to UV+ozone where the UV acts as the inline disinfection process, and the hydrogen peroxide serves as the strong oxidant by decomposing into hydroxyl radicals.^39,89^ The main difference from the UV+Cl_2_ systems is that for the UV+ozone and UV+H_2_O_2_ systems, only a portion of the swimming water is subjected to this effective treatment without a long-lasting residual chemical present.^29,39^ Without a high turnover rate of the swimming water, a traditional residual disinfectant like free chlorine is still almost always necessary when using these alternative systems.

There has been a lot of success with using UV in this market, although it remains quite expensive as a disinfection system for continuous treatment of swimming water when compared to other existing methods. For a hot tub, UV systems are becoming more popular as a manufactured add-on. Smaller lamps can be used in combination with chlorine or ozone to reduce the overall chemical demand and treat the smaller volumes of water more effectively. Other advantages of adding UV into the treatment system for swimming water is that UV works to inactivate some common chlorine-resistant parasites like *Cryptosporidium*.^43,50^ UV is also advantaged as an add-on process because it uses no chemicals, therefore minimizing the production of DBPs, making it safer than other alternatives.^29^ Unfortunately, in UV+Cl_2_ systems, some studies have shown that the addition of UV does indeed reduce the formation of some DBPs but may enhance the formation of others.^47−49^ Several studies have expressed concerns in byproduct formation (specifically chloroform, chlorinated phenols, and nitrogen containing N-DBPs) from the use of combined UV and chlorine disinfection as reviewed by Kimura et al.^96^ Combined UV+Cl_2_ was stated as a serious concern, as it was confirmed to produce higher levels of DBPs than the individual methods, and potentially result in higher water toxicity. Despite this, generally, people still consider UV disinfection (especially independently) to not produce significant levels of regulated DBPs and still a promising alternative technology to continue to implement in pools/spas.

According to the National Sanitation Foundation (NSF) through the American National Standards Institute (ANSI), the NSF/ANSI 55 requires UV disinfection intensity for pools/spas to be a minimum of 40 mJ/cm^2^ for Class A (contaminated water) or 16 mJ/cm^2^ for Class B (drinking water) in order to inactivate the pathogenic microorganisms that could be present.^97,98^ Studies have also previously shown a UV dosage of 1.34 kWh·m^–3^·d^–1^ was realistically applied to treat a public pool.^44,45^ In this pool, they determined the UV dosage required to remove free chlorine from the pool was only 0.22 kWh·m^–3^·d^–1^ and to remove 90% of combined chlorine was only 1.0 kWh·m^–3^·d^–1^.^44^ This means the UV light will continue to decompose free chlorine while active in a pool, resulting in UV treatment of chlorinated water to increase the overall chlorine demand and concentration needed to maintain a residual disinfectant.^99^ Despite this, UV has also shown a strong ability to reduce the combined chlorine concentration reducing the overall DBP formation in the same system. The remaining drawbacks of UV as a sole system will always be the lack of any residual disinfectant and its inability to provide oxidative power.^89^ Because of this, UV alone has little to no effect on the characteristics of the swimming water such as clarity and odor.^46^ Other major issues can arise from the turbidity of the water as this can reduce the effectiveness of UV light penetration.^39^ Similarly, a continued drawback of UV systems include the expensive cost of UV cells and lamps.^39^

### 2.6 Antimicrobial Metals

Antimicrobial metals have a long running history of applications to disinfect waterborne pathogens in swimming pools, hospitals, domestic hot water, and even drinking water due to their natural biocidal properties.^100−103^ These metals have gained more popularity in treatment of pools/spas over the last few decades and are now commonly used through many different systems and have been nicknamed as mineral treatments in the water recreation industry.^54^ The most common metals used are copper (Cu), silver (Ag), and zinc (Zn) and the main methods they are administered are through Cu/Ag ionization (CSI), Cu/Ag/Zn cartridges, and Cu-based algaecides.^39,54^ All methods of metal ion disinfection are most commonly used in addition or as a supplement to a primary disinfectant like traditional chlorine with the promise of lowering the overall residual chemical demand.^54^

CSI is administered through electrochemically generated Cu and Ag ions. This is most commonly achieved by applying voltage between two Cu/Ag electrodes and releasing the metal ions directly into the swimming water.^26^ Previous studies showed that lower free chlorine concentrations (0.4 mg/L) with the addition of CSI in a controlled pool system resulted in the same efficacy as when maintained with only free chlorine at higher residuals (>1 mg/L).^103^ These CSI systems have also demonstrated other advantages when working together with traditional chlorine. Researchers found combined CSI+Cl_2_ systems not only decreased the residual chlorine necessary but could also decrease the concentration of DBPs by 80% and the cytotoxicity of these DBPs by up to 70%.^26^ The other commonly used system to disperse metal ions is with cartridges through a method of controlled erosion, but this results in much lower concentrations and rates of dissolution than ionization (Figure 1e).^39^ Because of their intended purpose and simple use, the cost and lifespan of these products are usually lower and shorter than the ionizers which tend to be more expensive and long-term.^39^ The concentrations of released ions are not commonly disclosed by the products currently available on the market, but were shown in some studies to have very little and concerning effects on disinfection capacity when using the recommended low residual chlorine dosages.^39^ Cartridges are a simple and passive system that can be easily administered to pools/spas but are limited in their ability to produce strong and fast disinfection capacity due to the need for continuous turnover of the swimming water. Lastly, the common use for Cu in pools/spas is in algaecide products due to Cu’s ability to react and inhibit the growth of algae cells.^104^ Many previous studies have also concluded Cu to be the best additive for treating algae most commonly found in swimming waters with low concentrations <0.6 mg/L.^51,52^

As these antimicrobial metal-based products most often release low dosages of Cu, Ag, and/or Zn ions into the swimming water, there is no material health concern to the user as long as these concentrations remain within the health and safety standards. Unfortunately, these alternative solutions cannot often be used as a primary disinfectant in pools/spas as the low concentrations alone are not strong enough to upkeep the necessary residual antimicrobial power and deter potential waterborne pathogens.^39,54,105^ Some of these antimicrobial metal ions have been studied for their ability to inactivate and reduce transmission of chlorine-resistant pathogens like *Cryptosporidium*; finding some potential for Cu/Ag applications in pools/spas to reduce outbreaks.^43,53^ Although these metal ions have also been reported to enable users to lower their chlorine demand, which helps minimize harmful DBP formation, it does not eliminate it. Additionally, the usage of metal ions in pools/spas are all administered in passive processes, so there is limited ability to have any quick turnover of the entire water volume. Lastly, the most common problem users often face with metal ions in pools/spas is staining. Staining is an inevitable occurrence if the pools/spas are not strictly maintained since all the added metal will eventually precipitate from the water and deposit onto the surfaces. It has been reported that 30% of Ag and 10% of Cu ions added to the swimming water can be lost every day when using an ionizer.^39^ Cu specifically can cause the water to turn green and the surfaces to turn an unsightly green, blue, gray, and black, while Ag can cause brown and black staining, and Zn in excess concentrations can cause the water to become cloudy through precipitation of zinc carbonate.^39,54^ This is a common enough issue that manufacturers recommend users maintain a low Cu concentration of only 0.2–0.3 mg/L and a low Ag concentration <0.03 mg/L for example, to try and mitigate this issue.^39^ Manufacturers may also include stain removers in their products that use metal ions as a preventative measure.

## 3 Market Research Interviews

Our team conducted 100 market research interviews with various roles in the pool/spa industry. The breakdown of expertise for all individuals interviewed are shown in Table 2. Pool professionals (23) included pool/spa servicers, retail managers, and aquatics coordinators. C-suite representatives (14) included top ranking personnel like the chief executive officers, presidents, or vice presidents of specific pool/spa companies or disinfection technologies. Lastly, our largest interviewed group of sales and marketing representatives (37) included all ranks of knowledgeable personnel below the C-suite level who we spoke with regarding any manufacturing, distributing, or sales of pools, spas, and disinfection systems. Facility managers included pool operators and chief engineers of public or private facilities, like hotel pools/spas for example. These interviews were conducted face-to-face, and each took an average of 20–40 min. Questions regarding the individual’s position and daily responsibilities in respect to pools/spas were scribed. Questions about disinfection mechanisms and treatment approaches for the swimming water were asked for all interviewees in a nonbiased approach. Further questions were asked to gain experiences, general knowledge, and opinions on current existing methods and emerging technologies in the space. The results from all interviews were then organized and responses were coded for patterns specific to the methods used for treating and disinfecting swimming water. For further details on each step of our methodology for conducting interviews please refer to the Supporting Information (SI) document. Quantifiable metrics were also gathered through our 100 interviews and averaged together to compare the relative costs and lifetime ranges for each of the six different technologies in either a pool or spa (Table 3). Beyond this we found that most consumers group chlorine and bromine, and UV and ozone together as they are commonly associated with each other. Because of this, the following summarizes all the market research findings for each of the existing methods previously discussed in four main categories (chlorine/bromine, salt systems, UV/ozone, and antimicrobial metals).

**Table 2:** Breakdown of All 100 Individuals Interviewed by Relevant Position Type

Industry Roles/Titles	No.
Pool Builders/Construction	5
Pool Owners/End Users^a^	11
Pool Professionals	23
C-Suite^b^ Representatives	14
Facility Managers	10
Sales/Marketing Representatives	37
Total	**100**

a End Users refers to any swimmers/bathers that could actively use the pool/spa and be exposed to the water, chemicals, and care needed.

b Chief executive officers, presidents, or vice presidents of specific pool/spa companies or disinfection technologies.

**Table 3:** Relative Costs and Lifetimes for 6 Different Pool/Spa Disinfection Technologies^a^

	Pool^c^		Spa^c^		
Disinfection Systems^b^	Initial Cost ($)^d^	Operation and Maintenance ($/yr)^e^	Initial Cost ($)^d^	Operation and Maintenance ($/yr)^e^	Typical lifetime range^f^
Chlorination	30–500	400–1500	30–1800	136–408	7–10 years
Bromination	NA^g^	NA^g^	30–1800	136–204	7–10 years
Salt System	1000–2000	550–1300	2000–4200	400–600	2–5 years
Ozonation	600–5000	0	80–200	0	2–3 years
Ultraviolet Radiation	850–1800	600–1200	106–400	70–140	6–12 months
Antimicrobial Metals^h^	0	20–60	0	30–160	1–12 weeks

a The estimated initial costs, operation and maintenance costs, and typical lifetime of each system is summarized for the 6 categories of disinfection technologies: chlorine, bromine, salt systems, ozonator, UV, and antimicrobial metals.

b All values are an estimated cost range for the consumer determined from interviews conducted with 100 individuals in the pool/spa industry. Specifically, the interviews were obtained from various regions of the US and represent a diverse average from all areas. During our interview process, we would ask retailers, experts, and end users about the overall costs for running a system, average spending annually, time to replace a system, etc. All values were totaled, and the relative range is shown in the table.

c Pool/Spa specific cost ranges are determined for varying sizes and volumes typical for private use (homes, hotels, apartments, etc.). Commercial/Olympic size pools/spas are not included in this analysis.

d Initial cost refers to the disinfection system alone. No additional costs, maintenance, chemicals, cells, or initial dosages are included.

e Operation and maintenance (O&M) here refers to the estimated cost to a consumer for one year of use including necessary replacement chemicals, additional cells, initial dosages, and annual maintenance minus the associated initial costs.

f The typical lifetime values presented are for the range reported by both the product manufacturers and consumers who have experience using a specific technology. This information was gathered from consumers and product representatives during the 100 interviews collected.

g Bromine costs are not provided as it is uncommonly used for pools and no consumer data was obtained.

h For the antimicrobial metals example, the listed costs are representative for typical one-time use cartridges and mineral additions to an already chlorinated swimming pool/spa.

### 3.1 Chlorine and Bromine

**Figure 2:**
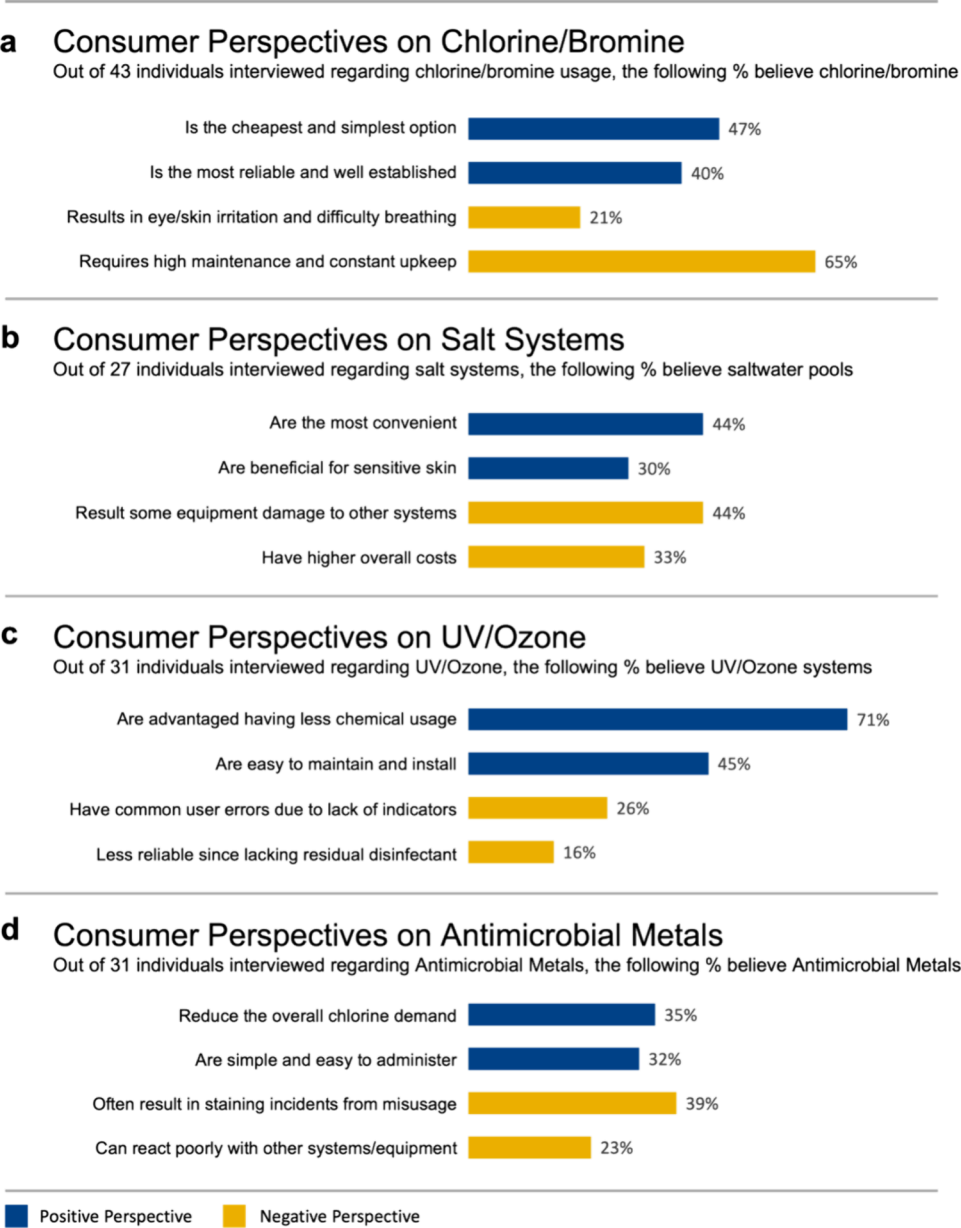
**Interview results for various disinfection methods, positive and negative public perspectives.** Consumer perspectives resulted from our 100 interviews are summarized for 4 main disinfection categories: (a) chlorine/bromine, (b) salt systems, (c) UV+ozone, and (d) antimicrobial metals. Each group shows the most mentioned opinions and experiences, both positive (blue) and negative (gold), regarding the cost, usage, or maintenance of each system. The data represents the percentage of individuals out of the number of interviewees that discussed a specific disinfection approach and are not out of the total 100 individuals as not all interviewees have experience with all the disinfection methods discussed. (For any reference to color in the figure, the reader is referred to the online/web version of this article).

Throughout all our interviews with professionals, a strong preference for chlorine/bromine continues to exist in the industry. With over 50 years of use, it is well-established and trusted by users, professionals, and organizations like NSF, EPA, and PHTA. From a consumer viewpoint of 43 interviewees that had experience with chlorine/bromine disinfection, 47% of users found chlorine/bromine-based systems for pools/spas to be generally inexpensive, straightforward, and more cost-effective compared to alternative disinfection methods available today (Figure 2a). 40% trust chlorine/bromine more than alternatives as it is a well-established method and more commonly available (Figure 2a). Chlorine and bromine’s main drawbacks are the generation of disinfection byproducts (DBPs), causing harm and sensitivities among users. Our research revealed that 21% of users reported sensitivities while using chlorinated/brominated pools and spas (Figure 2a). These sensitivities encompass various issues such as headaches, asthma, cough, itching, difficulty breathing, and stinging eyes. Lastly, 65% of users also reiterated the need for complex and consistent water balancing, or upkeep of water chemistry, to maintain a chlorinated/brominated pool/spa (Figure 2a). Maintaining a pool involves monitoring various chemical factors like pH, metals, hardness, and residual chlorine/bromine, ensuring they remain balanced. Outdoor pools face the additional challenge of faster chlorine depletion due to sunlight exposure. To address these concerns, regular testing of chlorine levels is essential, requiring daily/weekly assessments for effective pool maintenance. Balancing these factors is crucial to ensure a well-maintained and safe swimming environment. From our market research interviews, we learned that chlorine/bromine has remained the popular standard for pools/spas all these years because it has been a well-established method, cost-effective, and aided by the advantages of shocking. We also heard from many about the chlorine shortage that occurred in 2021 that resulted in the spike of chlorine prices and rise of alternative technologies in the industry to combat the shortage.

### 3.2 Salt Systems

Many professionals in the water recreation industry agree that saltwater pools and salt systems are not the best for long-term pool maintenance and costs but agree it is found more desirable by the end-user. 44% of users mentioned lower maintenance as the biggest motivator for switching (Figure 2b). This is because the salt systems are designed with convenience in mind, with a constant dosage of chlorine generated, and it does not need to be constantly added manually. Overall, our market interviews concluded salt systems are commonly used in hotel and resort pools to offer a low maintenance and luxury feel. The reduced chlorine concentration and lower daily/weekly maintenance create a more convenient experience for the user or owner or a pool/spa. When speaking with pool professionals regarding salt systems, it was discovered that many favored the alternative over traditional chlorine for the reported user-friendly benefits. Analyzing the interview data, 30% of respondents mentioned less eye irritation, smoother skin feel, and improved breathability in saltwater pools compared to those treated with traditional chlorine (Figure 2b). Many distinctly stated they prefer saltwater pools specifically due to eye, skin, and breathing sensitivities. The consistent chlorine production of salt systems also contributes to maintaining a stable and reliable free chlorine residual.

When interviewing professionals, 44% warned us about the potential disadvantages of salt systems including damage to other equipment in the pool and not actually being cost-effective down the line as the systems can often negatively react with other soft metals used in the construction or equipment of the pools (Figure 2b). There is a risk of corrosion and rust on metal surfaces and pool equipment that many pool professionals and end users reported. The increased salinity and use of electricity in these systems can cause damaging effects. Users and professionals have noticed that more maintenance and repairs may be needed in the long term, potentially adding to overall costs. Pool owners must assess factors like their pool lining, metal handlebars, ladders, and other equipment when considering a switch to these systems. All our interviews concluded that while salt is more economical than chlorine, salt systems come with a higher upfront cost, typically exceeding $1000 for the system alone. Additionally, replacing salt cells used in the electrolysis process every couple of years adds to the overall expense. 33% of users emphasized that it is not actually cheaper or more cost-effective than traditional chlorine (Figure 2b). Despite this, salt systems were the solution to chlorine without having to purchase chlorine as it produced it in situ. This made it very popular during the shortage.

### 3.3 UV/Ozone

According to our literature review and market research interviews, UV and ozone systems both individually produce much fewer residual chemicals in the swimming water and are often used in a combination/mixed approach (UV+ozone). We will mainly be referring to UV+ozone in this section as most interview responses referred to them as such. 71% of users are aware of the less chemical dosage as a big motivator for the installation and use of these systems either independently or combined (Figure 2c). UV+ozone systems are very easy to attach to most pools/spas, as they are designed to be directly integrated into the established water flow. Installation by servicers can take less than an hour, as 45% agree UV+ozone systems are easy to maintain and replace, reporting minimal maintenance requirements, occasional replacements, and nonfrequent cleanings (Figure 2c).

A drawback identified with these systems results from their passive nature, as users might not realize when the effectiveness of UV+ozone decreases. We found 26% reported some level of user error or lack of knowledge on the proper maintenance and replacement processes (Figure 2c). Ozone systems may stop working after a couple of years and UV lamps every 6 months to 1 year. Many professionals mentioned UV lamps become cloudy and permanently fogged after prolonged use, completely depleting the ability to function without light penetrating through the lamp. UV systems can also lose effectiveness over time due to dimming bulbs and calcium buildup on the lenses. Beyond this, manufacturers and sellers quoted the concerns from customers very rarely returning to replace the lamps after one year, thus leading us to conclude many users do not bother with the maintenance, upkeep, or expensive replacement of UV lamps in pools/spas; therefore, making it an unreliable disinfection solution for swim and leisure. We believe the replacement of the cells/lamps are often overlooked due to the lack of proper maintenance or obvious indicators. 16% of interviews also mentioned that both UV and ozone are disadvantaged by the lack of residual disinfectant neither produce (Figure 2c). UV itself leaves no residual disinfectant while ozone has a very short half-life limiting its residual capacity. Our market research also confirmed ozone is not commonly used in larger water bodies since a higher residual capacity is preferred, making ozone, by itself, a minimally impactful disinfectant in large pools.

### 3.4 Antimicrobial Metals

Mainly associated with Cu (copper), Ag (silver), and Zn (zinc), this category of disinfectants has been established mainly for the addition of these elements after a standard approach like chlorine or ozone is used. We have seen these metal/mineral specific products advertised in the market for years now, and according to our market interviews, they have gained tremendous popularity since consumers do not view these metal/mineral products with the same negative attitudes they may for traditional heavy chemicals. Our consumer analysis and market interviews give us confidence that customers think positively of these products and the use of “natural materials” in their recreational water as 35% mentioned the main driver being its ability to reduce the chlorine demand and decrease chemical usage in the swimming water (Figure 2d). Consumers also believe they are a more sustainable, eco-friendly, and safe form of treatment aiding in their growing popularity over the years.

These metals are particularly useful in combating chlorine-resistant microorganisms. Speaking with pool professionals, 32% approve of the method of delivering the metal ions through floating devices or cartridges, releasing the metals slowly to ensure consistent effectiveness (Figure 2d). Metals prove to be powerful disinfectants that can effectively remove such contaminants, significantly reducing the reliance on chlorine only. However, the primary drawback of introducing excess metals into the water is their potential to stain surfaces. Cu and Ag are known to cause stains on various materials, including pool/spa surfaces and equipment, 39% of users reported staining as their biggest issue (Figure 2d). The risk of staining is higher when these metals are present in abundance. Moreover, it is essential to note that Cu and Ag do not mix well with other pool systems. The combination of excess chlorine and metals can lead to more staining issues. Additionally, electrolysis systems, if used in conjunction with metals, can accelerate the erosion of these metals, diminishing their effectiveness as 23% percent of professionals mentioned this complication with other systems (Figure 2d).

## 4 Discussion

From our literature review of the various common disinfection technologies used to traditionally treat pools/spas, we have discovered almost all still contribute to DBP formation. Among the DBP concerns, many systems like UV+ozone, salt electrolysis, and antimicrobial metals aid in reducing the chlorine demand, thus reducing the overall DBP concentration and effects. DBPs are inevitably a side effect of using chlorine/bromine to disinfect pools/spas and we believe the EPA/CDC should play a stronger role in studying the effects of DBP exposure in pools/spas. The German standard DIN 19643 could potentially be a useful reference for US regulations to set stricter guidelines for DBP formation in swimming water.^1,79^ For this study focused on disinfection systems currently used in pools/spas, we were limited to the knowledge available in the literature and gathered through our face-to-face interviews, but future work should emphasize the untapped resources of business and market data available for several of these technologies. Analyzing the operating costs and maintenance for each disinfection system is considerably complicated as many are used in combination with one another resulting in confounding variables. Future work should consider the various elements that go into the potential costs for each system (energy requirement, lifespan, chemical costs, maintenance, replacement cells/bulbs, servicers/hired professionals, etc.). Because of the complexity and limited ability to interview individuals and receive cost relevant data, a more thorough cost analysis was determined to be beyond the scope of this study.

From our market research interviews we believe consumers are gaining awareness of the potential negative health effects from traditional chemicals like chlorine/bromine. With the COVID-19 pandemic behind us, we see increasing discussion around people’s individual safety, health, and sustainability. Through conversations with 100 individuals, we have concluded people are also applying this growing awareness and understanding to their swimming habits. This in combination with the massive chlorine shortage that occurred in 2021, there has been a tremendous increase in alternative solutions that reduce the chlorine demand or rid it altogether. The most important factors our market interview results suggest are (1) alternative solutions to reduce the free chlorine residual are gaining popularity rapidly, (2) both users and professionals value simplicity and well-established methods above completely new technologies, and (3) consumers in this industry value convenience and consistency above cost.

Research and development toward developing chemical-free technologies for pools/spas is very important as more and more people own their own pool/spa and children begin to learn to swim at consistently younger ages. Despite the potential hazards associated with chlorine-based solutions, it remains a dominant leader in the industry due to its well-established methods and ability to shock a large water body with quick turnaround. The current alternatives like UV, ozone, antimicrobial metals, and salt systems all classify as passive systems, that work continuously, but slowly. These passive systems lack a proper residual disinfectant, making disinfection turnaround time much longer as pools/spas are limited in their water circulation and flow rates. This is a particular area that research should focus on mitigating and improving in new and alternative technologies. Some emerging technologies that could have promising applications for improving water disinfection in swimming pools/spas are electrochemical processes, advanced oxidation processes, and some limited cases of cavitation bubbles. Specifically, researchers are developing new methods like locally enhanced electric field treatment (LEEFT) to disinfect water through smart pipes with low energy consumption and residual copper concentration to provide lasting antimicrobial power.^106−108^ This novel technology could have strong applications in pools and spas as it can provide both a low energy chemical system with electric field treatment, while supplying a residual antimicrobial metal concentration with copper that is lacking in many of the passive alternatives previously discussed.^109^ Future research directions should continue to focus on alternatives that continue to reduce the need for harmful chemicals in high dosages while still providing some level of residual disinfectant for the swimming water. This should be done through developing disinfection methods that can provide antimicrobial power both continuously and with faster turnaround time to mitigate the need for chlorine shock.

## 5 Conclusion

In this study, an overview of the literature on current disinfection systems used in pools/spa is reported. Emphasis was placed on the differences of these systems to one another and their overall contributions to DBP formations and reduction as reported in the literature. Face-to-face interviews were also conducted with 100 industry experts and end users in the US to gain a consumer perspective and understanding of trends in the market. The findings reassure a large increase in alternative technologies supported both in the literature reports and by the pool/spa professionals we spoke with. Overall, we conclude the future of water recreation is trending away from high dosage chlorine-based solutions to disinfect swimming water and turning to alternatives with more sustainability and safety in mind. The consumer’s perspective for convenience, luxury, and comfort is widening the market for new and improved technologies to step in and continue to reduce the negative effects of traditional chemical-based disinfection. Future work in the academic space can focus around further discussion on DBPs and the reduction through new and emerging technologies to disinfect water, while in the industry/market sector, more interviews to focus on the end users and their important values are key to ensuring a new technology can have the optimum impact. Other future directions for this research can include interviewing a number of scientists and academics in the space to gain their perspectives as researchers, as well as comparing all of the US data obtained in this study with data from other countries to understand how disinfection of pools/spas, costs, regulations, and values to the end users vary across the globe.

## Supporting Information Available

Additional details regarding the methodology behind the market research interviews (PDF)
