# Supplementary material for: Water Disinfection Systems for Pools and Spas: Advantages, Disadvantages, and Consumer Views in the US
Source: ACS ES T Water. 2025 Jan 9;5(2):525–38. doi: 10.1021/acsestwater.4c00612 (PMC11833866; doi:10.1021/acsestwater.4c00612)
Supplement: Supplementary file 1 — ew4c00612_si_001.pdf [file ew4c00612_si_001.pdf]

Supplemental Information

Water Disinfection Systems for Pools and Spas: Advantages, Disadvantages, and Consumer Views in the US

Mourin Jarin[1], Jackie Ly[1], Jonathan Goldman[2], Xing Xie[1*]

[1]School of Civil and Environmental Engineering, Georgia Institute of Technology, Atlanta, Georgia 30332, United States

[2]Office of Commercialization, Georgia Institute of Technology, Atlanta, Georgia, 30332, United States

[*]Corresponding author: Xing Xie, Email: xing.xie@ce.gatech.edu, Phone: 4048949723

1. Market Research Interview Methodology

For further context to the methodology behind the market research and interviews presented in this study, we have conducted research with industry professionals, specifically, market and customer centered interviews funded through the NSF I-Corps and Georgia Research Alliance grants. These grants are specifically allocated for institutional efforts to research and better understand the industry and market that our lab-based technologies can impact. After several years of market research, we have decided to summarize our findings as introducing an industry perspective that we believe can be very relevant and useful to the academic field. Our focus in this paper is to provide a general literature background on the most commonly used disinfection mechanisms in the industry in order to make the understanding of the market research easier to grasp. The scope is only pertaining to the current state of the industry as we found it through our face-to-face interviews with 100 professionals and current users in this field in the US. Because of this, topics like detailed DBP formation pathways and novel/emerging disinfection techniques are only covered in brief as it is not yet discussed in detail in the industry. In order to keep the focus still niche, we have maintained the scope of the paper to only what is currently found in the pool/spa industry in the US.

1.1 Grant and exception of IRB

In this study, no IRB approval was required or obtained. The market research interviews conducted were funded through the NSF I-Corps and Georgia Research Alliance (GRA) grants, both based in commercialization and market research efforts for institutional research. As these interviews focused on understanding market needs rather than gathering sensitive or personal data, IRB oversight was deemed unnecessary. The I-Corps and GRA programs explicitly exempt this type

of market research from IRB requirements. The interviews conducted as part of these studies followed all NSF I-Corps and GRA protocols, which are intended for customer discovery and market research purposes. There are no IRBs required for such market research and the interviews were conducted with experts, users, and mainly professionals in the respective field with verbal/written consent obtained for the interview and information collected.

1.2 Interview protocol

The lean startup methodology for conducting customer discovery interviews is the main basis for how we conducted our interviews and how we limited bias across the interviews and standardized the process for all the individuals who conducted interviews. The interview protocol followed the NSF I-Corps methodology, focusing on identifying customer needs, pain points, and areas of market opportunities for new technologies. We did not have a standardized questionnaire for all individuals since this would tremendously limit the variety of information obtained, but we did use a template/guideline/prompt for all interviews in order to ask similar questions to help guide conversation in each interview and obtain non-biased information in each case (shown below). These questions were tailored to the specific industry and market segment of water disinfection technologies.

Interview Notes for Example

<u>Interviewee information:</u>

Name: Company:

Title: Email:

Ask for consent to:

1. write/take notes on responses
2. contact again if have further questions
3. use information provided anonymously for any academic research, reports, or publication
4. make note in their interview information above if they do not agree or are not comfortable with their information retained for any purpose

Background on individual:

1. So tell me a little more about your position, what does a regular day look like for you?
2. Tell me more about XXX you mentioned, what does that process look like? How does that work?
3. Could you tell me more about XXX technology?
4. When was the last time you experienced using this system?

Product General Information:

1. Is it alright if I ask about some costs or maintenance you experienced regarding XXX?
2. Pool or hot tub? Size? Volume?
3. Capital costs? Maintenance cost? Regular maintenance needs?
4. Lifetime?
5. Any other opinions or thoughts you have on XXX?

Disinfection System:

1. Can I ask about some of the specific processes or please tell me more about how they work (if they are knowledgeable/professional)?
2. Ask about circulation flow rate? General treatment method? Any filtration or adsorption?
3. Disinfection method?
    a. If chlorine for example, ask about concentration, dosage, frequency, cost?
    b. If UV for example, ask about intensity, replacement frequency, cost?
4. Are these methods good enough in their opinion? Any issues or concerns on cost, corrosion, or human health?

Thoughts or expectations about new water disinfection technology:

1. Heard about alternative or trending new systems in the market? Which if any?
    a. Any comment on it? Pros & cons? Thoughts?
2. Do you have experience working with any alternative or new systems?
3. Or if not, what have you heard about them? Do you know anyone that has used them? What was their experience?

1.3 Participant selection

The two grants we used support industry level interviews with mainly users and professionals in their respective field. Because of this, participants were selected using purposive sampling, targeting individuals and organizations directly involved in or knowledgeable about the pool/spa water disinfection market. This included end-users, decision-makers, industrial players, and technology experts. There were no strict inclusion or exclusion criteria beyond relevance to the market studied.

Beyond this, the interviews were obtained through multiple years and with travel to different regions of the US for various industry conferences, expos, and even for singular interviews in some cases. Virtual interviews were conducted from anywhere in the US as well, while local interviews in our state of Georgia and city of Atlanta were also obtained. Because of this, we firmly believe we have secured a perspective that is diverse across the entirely of the US mainland.

1.4 Data analysis

Data from the interviews were analyzed qualitatively using thematic coding to identify common trends and insights about the market's needs and challenges. No statistical analysis was applied, as the focus was on extracting qualitative insights to inform present/future trends and areas of development in the pool/spa water disinfection industry.

1.5 Consent and confidentiality

Consent was obtained verbally or in writing from each interviewee before proceeding with the interview. As 80% of interviews occurred in person, consent was obtained for note taking during

the interview beforehand. For the 20% of virtual interviews, consent for note taking was obtained similarly beforehand or otherwise for recording/transcribing of the call. Following this, we receive consent from each individual either verbally or in writing if they approve us using the information provided for any such academic research, report, or publication. As the interviews were focused on general business and market information, no personal or sensitive data was collected. Participants were assured that their responses would remain anonymous and used solely for the purposes of this study. In respect to the paper, none of the names or relevant workplaces for each individual will be provided in order to maintain confidentiality of the interviewees.

1.6 Potential biases

There was a total of 6 individuals over the last 3 years who helped to conduct all 100 interviews and all individuals were trained through the lean startup methodology of customer discovery for non-bias approaches to interviewing industry experts and users in the field. This methodology is the main basis for how we conducted our interviews and how we limited bias across the interviews, as well as standardized the process for all the individuals who conducted interviews. We recognize that purposive sampling may introduce selection bias, as interviewees were chosen based on relevance to the market. To further mitigate this, we sought input from a diverse range of stakeholders, including those with varying perspectives on each aspect of the industry.
